# Supplementary material for: Synergistic Effects of Silicate-Platelet Supporting Ag and ZnO, Offering High Antibacterial Activity and Low Cytotoxicity
Source: Int J Mol Sci. 2023 Apr 10;24(8):7024. doi: 10.3390/ijms24087024 (PMC10138669; doi:10.3390/ijms24087024)
Supplement: Supplementary file 1 [file ijms-24-07024-s001.zip › ijms-2267456-supplementary.pdf]

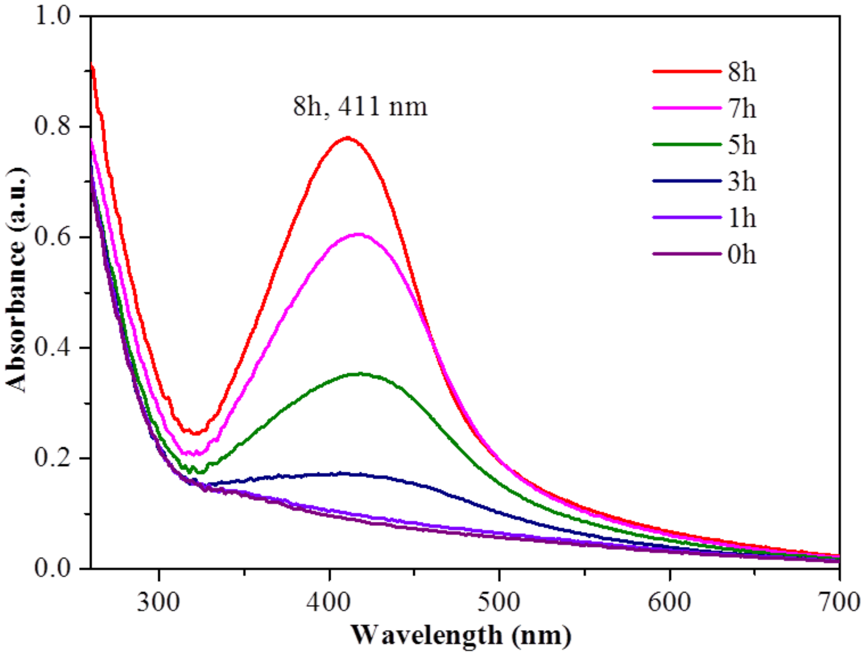

Figure S1. Kinetic measurements of nanoparticle formation at 411 nm UV-Vis absorption for AgNP/NSP (1/99, w/w) during the reduction of $AgNO_3$.

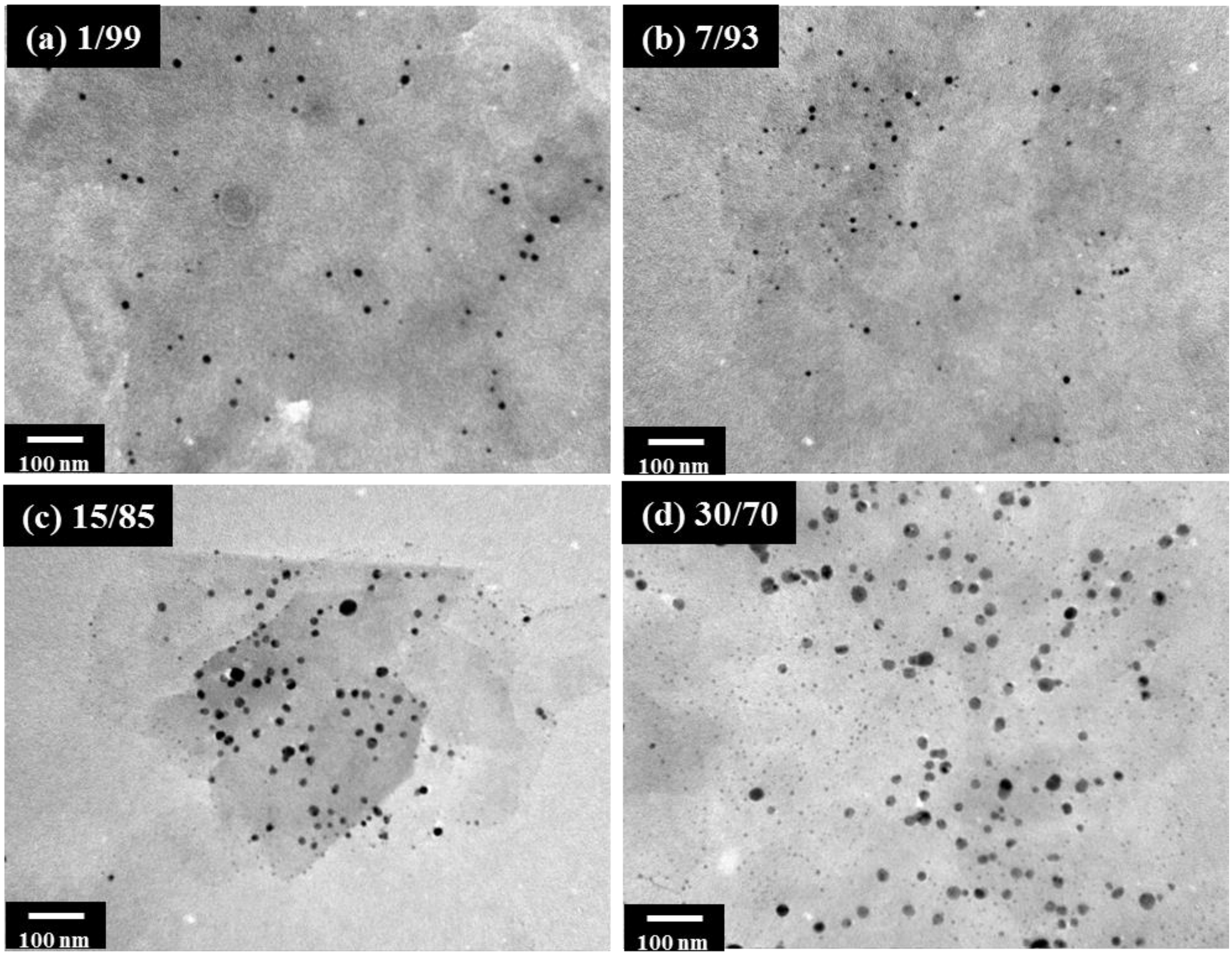

Figure S2. TEM images of AgNP and NSP in the AgNP/NSP hybrids at weight ratios of (a) 1/99 (b) 7/93 (c) 15/85 (d) 30/70

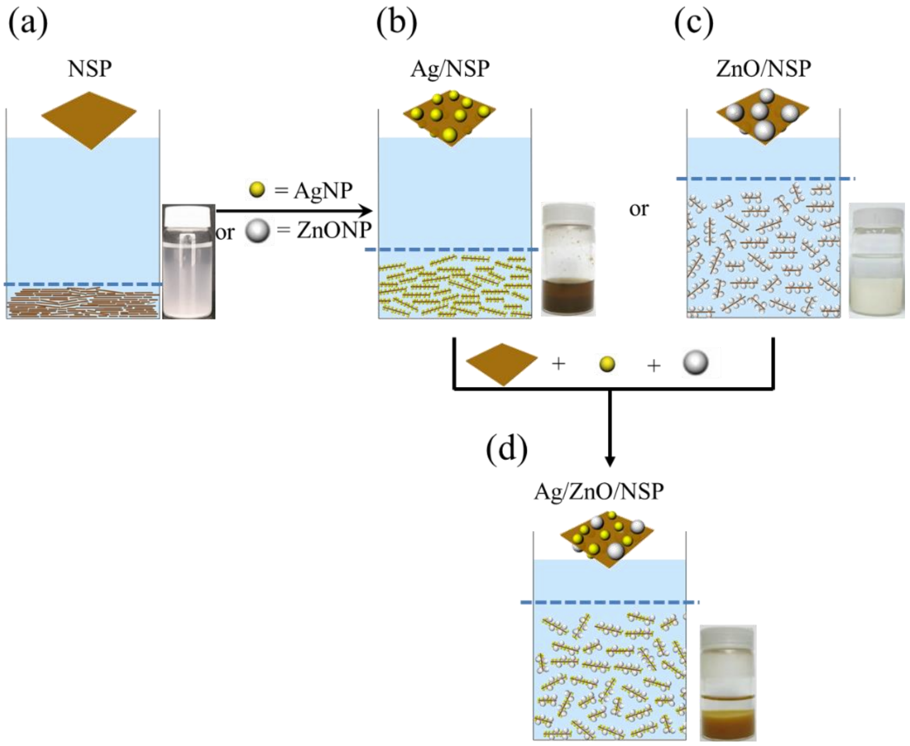

Figure S3. Conceptual illustration of (a) NSP, (b) Ag/NSP, (c) ZnO/NSP, (d) Ag/ZnO/NSP after resting for 3h.

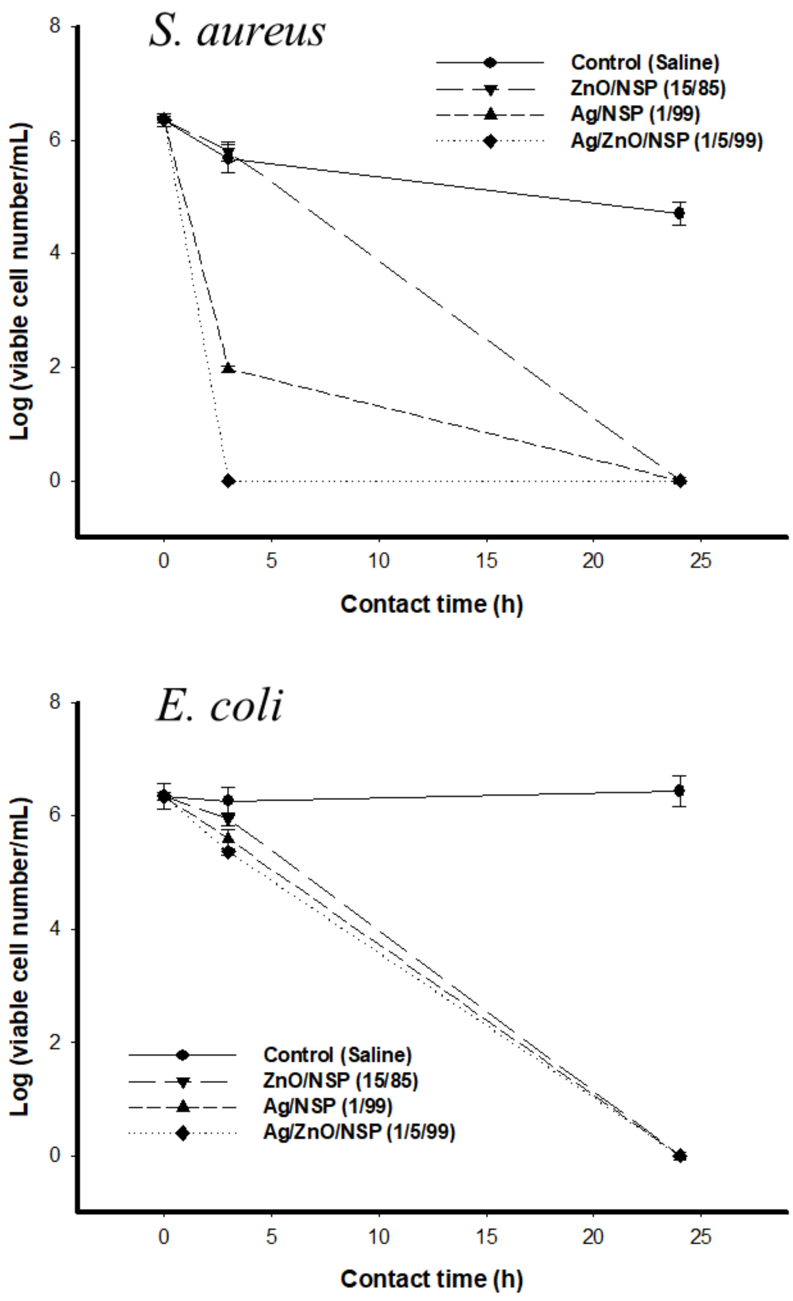

Figure S4. Antibacterial efficacies against *S. aureus* (Top) and *E. coli* (Bottom) in contact with three selected hybrids at their minimum bactericidal concentrations (MBC) for 0-24 h. The antibacterial efficacy: AgNP/ZnONP/NSP > AgNP/NSP > ZnONP/NSP.
